# Breast cancer stem cells

**DOI:** 10.3389/fphys.2013.00225

**Published:** 2013-08-27

**Authors:** Thomas W. Owens, Matthew J. Naylor

**Affiliations:** Discipline of Physiology, School of Medical Sciences and Bosch Institute, The University of SydneySydney, NSW, Australia

**Keywords:** breast cancer, cancer stems cells, transcription factors, cell fate, mammary gland

## Abstract

Cancer metastasis, resistance to therapies and disease recurrence are significant hurdles to successful treatment of breast cancer. Identifying mechanisms by which cancer spreads, survives treatment regimes and regenerates more aggressive tumors are critical to improving patient survival. Substantial evidence gathered over the last 10 years suggests that breast cancer progression and recurrence is supported by cancer stem cells (CSCs). Understanding how CSCs form and how they contribute to the pathology of breast cancer will greatly aid the pursuit of novel therapies targeted at eliminating these cells. This review will summarize what is currently known about the origins of breast CSCs, their role in disease progression and ways in which they may be targeted therapeutically.

## Introduction

Breast cancer is the leading cause of cancer death in women, causing extensive morbidity and psychological distress to millions globally. Encouragingly, the combination of better screening and treatment programmes have moderately improved the chances of surviving the disease, but there is still much to be done if the many women who are refractory to current therapies are to have a better chance of survival. Over the last decade breast cancer cells with stem-cell-like properties have been identified and characterized. There is now much interest around the role that these breast cancer stem cells (CSCs) have in the disease and whether they provide the key to unlocking new insight into the mechanisms driving breast cancer progression, drug resistance and reoccurrence.

Often described as a caricature of normal tissue development, cancer occurs when the regulation of tissue homeostasis is perturbed, resulting in the evolution of cells with increased growth and survival potential. The breast, like many other organs, is a hierarchically-organized tissue maintained by a series of stem and progenitor cells that have decreasing potency as they differentiate toward terminally-committed epithelial cells. Below, we describe briefly the normal breast epithelial hierarchy, but for comprehensive analyses we recommend (Visvader, 2009; Van Keymeulen et al., 2011; Raouf et al., 2012; Šale et al., 2013).

The breast is composed of a bilayered epithelium comprising two main epithelial cell types; luminal and basal (Watson and Khaled, 2008; Gusterson and Stein, 2012). The luminal cells line the ductal structures that will transport milk to the nipple during lactation. The basal cells surround the luminal cells and are in contact with the surrounding basement membrane that separates the parachyme from the stromal component of the tissue. Mammary stem cells (MaSCs) share cell surface and expression profiles consistent with basal cells and are hence thought to reside within the basal compartment of the gland. Isolated several years ago through the use of cell surface expression markers, cell populations greatly enriched for MSCs have been shown to be capable of reconstituting an entire mammary gland when transplanted into a mammary fat pad cleared of endogenous epithelium. Furthermore, serial transplants have demonstrated that the MSCs can self-renew as well as give rise to the other cell types (Shackleton et al., 2006; Stingl et al., 2006).

Initially thought to be restricted to relatively few cell types (luminal, basal, and stem cells), the repertoire of mammary cell types has expanded over the last few years. Development of lineage-specific markers and *in vitro* functional assays has enabled the isolation of discrete sub-populations of epithelial progenitors (Raouf et al., 2012; Sheta et al., 2012). Using an alternative approach, *in vivo* lineage-tracing has recently identified previously undescribed epithelial cell types (Šale et al., 2013). In the future, these techniques will likely unearth additional levels of complexity in the epithelial cell hierarchy that will no doubt aid our understanding of breast cancer and CSCs. However, when discussing CSCs, it is imperative to highlight that they are distinct from normal stem cells.

## Defining cancer stem cells

It is important to clarify that although they share functional similarities to normal stem cells, CSCs are not necessarily derived from stem cells. A CSC is functionally defined by the ability to (1) form a tumor in immunocompromised mice, (2) self-renew—shown by tumor formation in secondary mice and (3) “differentiate,” i.e., produce cells with non-stem cell characteristics (McDermott and Wicha, 2010).

In certain tissues, new technological advances are enabling CSCs to be studied in their primary setting, without the need for transplantation, however comparable studies have not yet been described in the breast (Chen et al., 2012; Driessens et al., 2012; Schepers et al., 2012).

We have chosen to use the term CSC but we recognize that cells with defining features of CSCs are also referred to as tumor-initiating cells (TICs) and tumor-propagating cells. In the majority of cases, these terms refer to the same functional entity. TICs can also describe the cell from which the cancer originated and CSCs may form long after the tumor was initiated. The cancer cell of origin is discussed in length elsewhere (Visvader, 2011). This review will focus on breast CSCs, their origins, pathological significance and potential therapeutic strategies to tackle them.

## Discovery of breast cancer stem cells

Historically, the hematopoietic field has led the way in the identification of stem and progenitor cells and their resulting lineages. The same was true in the CSC field, with the CSC-theory in solid tumors validated only relatively recently (Al-Hajj et al., 2003). Using cell surface markers Al-Hajj and colleagues found that CD44^+^CD24^−/low^ Lin^−^ cells from breast cancer patients were significantly enriched for tumor forming ability in NOD/SCID mice compared with CD44^+^CD24^+^ Lin^−^ cells. Moreover, the tumors formed by CD44^+^CD24^−/low^ Lin^−^ cells could be serial passaged (self-renew) and also reproduce the tumor cellular heterogeneity observed in the initial tumor (differentiation).

CD44 is a cell surface receptor for the extracellular matrix molecule hyaluronan, that influences cell behavior by direct signaling/structural roles or by acting as a co-receptor for receptor tyrosine kinases (Ponta et al., 2003). CD24 is a cell surface glycoprotein whose level of expression has become commonly used to isolate distinct cell populations from the normal mammary gland and breast cancer cells. CD24^*high*^ expression in normal human mammary gland and breast carcinoma corresponds to a differentiated gene expression signature, whereas, CD44^+^ cells exhibit a more “stem-like” signature of gene expression (Shipitsin et al., 2007). In the mouse mammary gland, CD24^−^, CD24^low^, and CD24^high^ expression levels correspond to populations of non-epithelial, basal and luminal epithelial cells, respectively (Sleeman et al., 2006). Functionally, the epithelial cell populations exhibited differential stem potential in mammary fat pad transplantation assays, with CD24^low^ cells being significantly enriched for mammary gland repopulating capacity.

The combination of CD44 and CD24 expression have been used to successfully enrich for CSCs in both cell line and tumor samples but caution must be exercised. For example, within epithelial populations CD44^high^CD24^−^ was shown to mark mesenchymal-like cells that formed mammospheres and had an invasive phenotype, but the cells lacked the capacity to produce the heterogeneity of the parental cell line (Sarrio et al., 2012). Therefore, these cells did not meet all the criteria of bona fide CSCs and thus highlight the importance of functionally testing “stemness” rather than assuming that a particular combination of cell surface markers is indicative of a phenotype.

In addition to cell surface markers, other expression-based methods of CSC-enrichment have been developed. Aldehyde dehydrogenase (ALDH) activity has been identified as a method of enriching for normal human breast stem and CSCs (Ginestier et al., 2007). Furthermore, by combining ALDH activity with CD44^high^CD24^−^ expression, the CSC fraction was refined further compared to either method alone. Interestingly, the ALDH^−^, CD44^high^CD24^−^ population was not enriched for CSCs demonstrating that the CD44^high^CD24^−^ population retains significant heterogeneity.

Separating cell populations based on protein expression profiles of either cell surface markers or ALDH1 requires functional validation of the isolated cells to confirm their capacity as CSCs. Recently, Pece and colleagues developed a novel reciprocal approach of using function to isolate CSCs that were then used to identify new markers. By taking advantage of the stem cell ability to survive in suspension culture combined with slow proliferation rate they isolated stem cells from normal human mammary gland based on retention of a membrane-labeling dye, PKH26 (Pece et al., 2010). Gene expression analysis of the PKH26^+^ cells revealed a novel set of stem cell markers that the group then used to isolate stem cells from both normal breast and tumor samples (i.e., DNER and DLL1).

Due to the intra- and inter-tumor heterogeneity in cancer, it is possible that CSCs from different tumors have distinct expression profiles. Thus, isolating CSCs by function and detailing their expression profiles may prove extremely valuable where traditional markers fail.

## Origins of cancer stem cells

**Figure 1:**
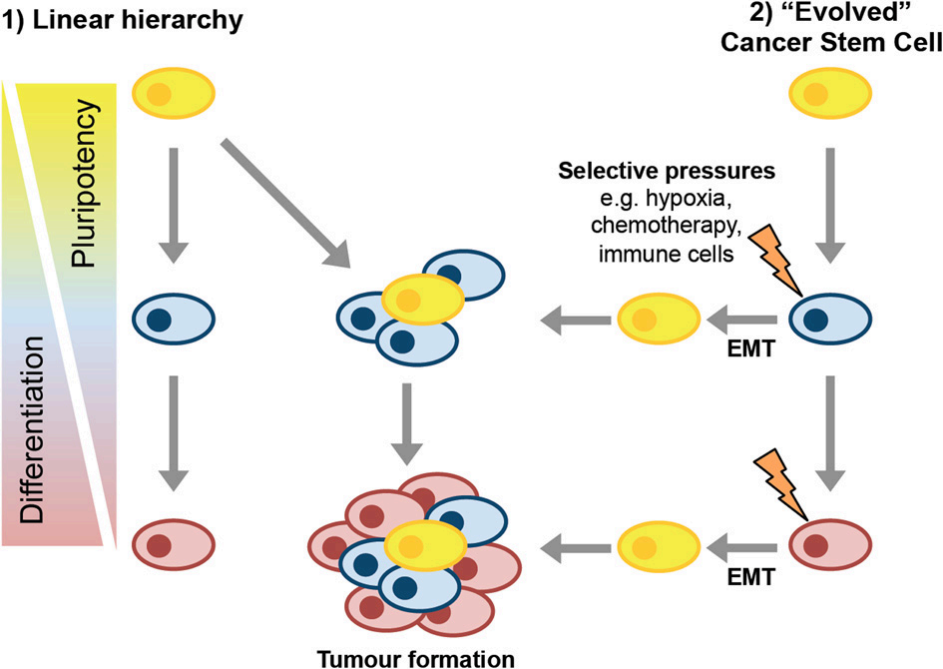
**Models of CSC formation.** In the linear hierarchy model of CSC formation, the transformation events that drive tumorigenesis occur in a stem or progenitor cell that then gives rise to more differentiated progeny as the tumor develops. These differentiated progeny have reduced tumor-forming potential. In the second model, cancer stem cells evolve, perhaps via induction of EMT, either as part of disease progression or in response to selective pressures in the tumor microenvironment.

The stem cell characteristics of CSCs draw in to question the cell type from which they derive. Two potential models of CSC formation are: (1) the tumor cell of origin had stem cell or progenitor properties, or (2) the tumorigenesis process yields cells distinct from the cells of origin that are capable of reconstituting the tumor (Figure 1).

The simple model of hierarchical tissue organization suggests that as cells differentiate along a particular lineage, they lose the potential to give rise to multiple cell types and are therefore less likely to be able to act as CSCs. Normal stem cells already have many of the properties associated with CSCs. Moreover, the long-lived nature of stem cells allows more time for multiple genetic lesions to be acquired. Therefore, it is possible that CSCs originate from tissue stem cells.

Studies demonstrating an increased risk of breast cancer in children exposed to radiation suggest that the cells subject to transformation would be long-lived stem or progenitor cells (Miller et al., 1989; Modan et al., 1989). Much more recently, luminal progenitor cells were identified as the likely cell of origin in *BRCA1* driven tumors (Lim et al., 2009; Molyneux et al., 2010; Proia et al., 2011). Cells displaying the markers of stem cells have also been identified in early DCIS lesions suggesting that possible CSC are present at early stages of tumorigenesis (Pece et al., 2010). If the transformed cell has stem/progenitor properties then it is understandable that this could give rise to CSCs, as well as the non-CSCs that make up the majority of the tumor.

The model in which the cancer cell of origin is responsible for the properties of the CSC would be encouraging when it comes to designing therapies to tackle the disease. If the tumor behaves in a rigid linear hierarchy with relatively few stem cells giving rise to the majority “differentiated” tumor cells then therapies that can kill CSCs or drive them to differentiate would remove the ability of the tumor to regenerate following therapy.

However, cancer is a disease that forms over many years, so even if the original transformation event had occurred in a stem-like cell, the tumor that presents at the clinic is likely to be a much more evolved and heterogeneous entity than a linearly-hierarchical tissue. A linear hierarchy in cancer would also not explain why recurring tumors are resistant to therapy, as successive rounds of tumor growth may be expected to be produce similarly-sensitive progeny. In this sense, it appears that tumors have also evolved mechanisms to be self-sustaining even if their original CSC pool is destroyed, potentially via the generation CSCs cells from non-stem cells.

## Formation of CSCs from non-CSCs

A range of breast cancer cell lines are now known to be composed of a heterogeneous mixture of cells. A proportion of the cells act as CSCs by being able to give rise to all the cell types within that line, while the other cells show reduced ability to act as CSCs. There is also suggestion of heterogeneity within the CSC populations themselves (Wong et al., 2012). Significantly, several studies have now demonstrated that cells have the capacity to interconvert between phenotypes.

Breast cancer cell lines SUM159 and SUM149 sorted into stem-like, basal and luminal populations demonstrated the ability to transition between these cell states to maintain the overall heterogeneity of the parental line (Gupta et al., 2011). This stochastic cell state transition enabled purified populations to reconstitute the proportions of the parental cell line within 11 days of sorting (Gupta et al., 2011). Piggott and colleagues used the mammosphere assay to demonstrate that MDA-MB-231, BT474, SKBR3, and MCF7 cells all contained self-renewing mammosphere forming units (MFUs). Interestingly, BT474 cells depleted of MFUs reacquired these progenitor-like cells following 4 weeks in culture (Piggott et al., 2011). *In vitro*, Ca1a, MCF7, Sum159, and MDA-MB-231 breast cancer lines, sorted CD44^+^CD24^+^ non-invasive cells could give rise to invasive CD44^+^CD24^−^ cells (and vice versa), even when initially plated as single cell clones (Meyer et al., 2009).

The generation of CSCs from non-CSCs has been confirmed *in vivo* using transplantation assays. Clones of non-invasive CD44^+^CD24^+^ sorted cells from Ca1a, ZR75.1 and MCF7 breast cancer lines transplanted into immunocompromised mice gave rise to molecularly heterogeneous tumors that exhibited local invasion (Meyer et al., 2009). Moreover, the stem-like-depleted basal and luminal populations of SUM159 cells were also able to transition to stem-like cells during tumor formation in NOD/SCID mice. However, it is interesting that the non-stem-like SUM159 populations required co-injection with irradiated parental SUM159 cells for tumor formation to occur. This co-injection requirement suggests that additional factors to those in the homogenous luminal or basal populations are required for conversion to stem-like phenotypes (Gupta et al., 2011).

Recent evidence suggests that the ability of the cancer cells to trans-differentiate is related to the transformation process. Using an inducible Src oncogene to drive transformation of MCF10A cells, CSC-like cells were generated during the transformation process within 16–24 h of Src activation (Iliopoulos et al., 2011). Furthermore, once generated the relative proportion of CSCs was maintained over several weeks in culture. Isolated CSCs readily formed non-CSCs whereas the reciprocal spontaneous conversion did not occur. However, media from CSC was found to drive non-CSCs to form CSCs and this was dependent of IL-6 (Iliopoulos et al., 2011).

Chaffer and colleagues demonstrated that hTERT-immortalized HMECs gave rise to a population of floating cells they term HME-flopcs (Chaffer et al., 2011) CD44^low^ HME-flopcs were able to spontaneously convert to CD44^high^ cells that had stem-like properties. Moreover, transformation of the HME-flopcs with the SV40 and H-ras increased the efficiency with which the conversion to CD44^high^ cells occurred.

Despite the growing evidence of the ability of non-CSCs to produce CSCs it is noteworthy that in the parental populations the proportions of CSCs remains constant over time. Even when sorted into distinct populations, the sorted cells eventually recapitulate the proportions of cells originally present in the parental line. Tumor molecular expression profiles remain constant during disease progression, suggesting a level of stability within a population of tumor cells (Ma et al., 2003; Weigelt et al., 2003). Moreover, similar molecular profiles of primary tumor and metastases suggest ancestors are common rather than genetically distinct (Sorlie, 2004). This supports a hypothesis that perhaps paracrine signals mediate a level of homeostatic control over the proportions of different cell types present within a tumor.

## CSC and epithelial-to-mesenchymal transition

Inter-conversion of CSC and non-CSC (spontaneously or otherwise) means that CSCs do not behave like classical stem cells. The question remains of how CSCs could arise from non-CSCs. Epithelial-to-Mesenchymal transition (EMT) is a natural process that occurs during development and is a method by which cancer cells metastasize during cancer progression (Thiery and Sleeman, 2006). EMT is also thought to be a mechanism by which CSCs form.

Induction of EMT in normal human mammary epithelial (HMLE) cells by expression of Snail, Twist or treatment with TGFβ1 caused the majority of cells to adopt the CD44^+^CD24^low^ expression profile consistent with CSCs. There was also a significant increase in the number of mammosphere forming cells following EMT (Mani et al., 2008; Morel et al., 2008). In addition to EMT driving cells to acquire stem cell characteristics, naturally occurring stem cell fractions of normal mouse and human mammary epithelium cells as well as human neoplastic samples expressed significant levels of EMT markers (Mani et al., 2008).

The mechanism by which EMT induces CSC formation may involve the transcription factor FOXC2, which was upregulated in immortalized normal human mammary epithelial (HMLE) cells in response to multiple EMT-inducing stimuli (Mani et al., 2007). The CSC-characteristics acquired through EMT were attenuated by suppression of FOXC2 expression (Hollier et al., 2013). Furthermore, FOXC2 was upregulated in CSC-enriched populations and expression of FOXC2 in V12H-Ras-transformed HMLE cells was sufficient to drive EMT and increase their tumor forming and metastatic potential in transplants (van Vlerken et al., 2013).

The ability of EMT-driving factors to induce CSC formation is likely to be dependent on the cell type in which EMT occurs. Slug is a transcription factor that can drive EMT and its expression is enriched in MaSCs. Exogenous expression of SLUG in luminal progenitor cells was sufficient to drive them to a more stem-like phenotype, whereas SLUG expression in differentiated luminal cells failed to do so (Guo et al., 2012). Interestingly, co-expression of Sox9 with Slug could induce differentiated luminal cells into a stem-like state by activating distinct gene sets. Moreover, Snail, but not Twist could substitute for Slug and cooperate with Sox9 in driving differentiated luminal cells into stem-like cells. Therefore, EMT contributes to, but is not sufficient for the non-stem cell to stem-cell transition and not all EMT-driving factors elicit the same effect (Guo et al., 2012).

Analysis of non-tumorigenic mammary epithelial cell lines (MCF12A, MCF10-2A, and MCF10A) and immortalized Myo1089 cells using EpCAM and CD49f expression levels, identified heterogeneous cell populations. The EpCAM^+^CD49f^+^ had an epithelial morphology with an expression profile characteristic of luminal progenitors, while EpCAM^−^CD49f^med/low^ were fibroblastic in appearance and expressed genes associated with EMT (Twist1/2 and Slug) (Sarrio et al., 2012). Interestingly, although the epithelial (EpCAM^+^) Myo1089 cells gave rise to mesenchymal-like cells that were more invasive and could form mammospheres, it was the epithelial cells that had higher ALDH1 activity and could recapitulate the heterogeneous cell populations seen in the parental line. Therefore, in this instance EMT was associated with a loss of stem-cell capacity and re-iterates the importance of determining “stemness” functionally (Sarrio et al., 2012).

The reprogramming of cancer cells into CSCs by EMT-associated transcription factors highlights the importance of understanding how transcription factor networks regulate cell fate determination in breast cancer (Kalyuga et al., 2012). The power of transcription factor-mediated cell fate control is most notably demonstrated by the creation of induced pluripotency stem (iPS) cells by the introduction of Oct4, Sox2, c-Myc and Klf4 into differentiated adult cells (Takahashi and Yamanaka, 2006). The same factors that induce pluripotency in normal differentiated cells may also be involved in the formation of CSCs. Non-tumorigenic MCF10A cells transduced with Oct4, Sox2, c-Myc, and Klf4 formed iPS-like cells that upon differentiation adopted a CSC phenotype (Nishi et al., 2013). These induced CSC-like-10A cells were largely CD44^+^CD24^low^, expressed ALDH1 and had high tumorigenicity *in vivo*. In metastatic breast cancer cells, Klf-4 expression increased the proportions of CD44^+^CD24^low^ and mammosphere-forming cells (Okuda et al., 2013). Oct4 alone was able to transform primary HMLE cells into cells capable of initiating tumors in xenografts and Oct4 is also thought to be the downstream effector of IL-6 induced CSC formation (Beltran et al., 2011; Kim et al., 2013).

Transcription factors mediate changes in gene expression, but the action of transcription factors is also influenced through epigenetic genome modification. Epigenetic regulation of gene expression controls cell fate specification by activating or repressing genes associated with lineage commitment. Epigenetic changes are also associated with cancer progression.

In mammary epithelial cells, repressive and activating histone methylation patterns are associated with changes in gene expression during lineage determination (Pal et al., 2013). CSCs isolated from breast cancer cell lines had elevated levels of the polycomb group protein, EZH2, which catalyses histone methylation (van Vlerken et al., 2013). EZH2 knockdown by siRNA moderately reduced the CSC populations in breast and pancreatic cancer cell lines, inducing a more differentiated pattern of gene expression. Moreover, high EZH2 expression correlates with poor prognosis in breast and prostate cancer (Varambally et al., 2002; Pietersen et al., 2008).

Interestingly, the methylation patterns in mammary epithelial cells alter during pregnancy and also in ovariectomized mice, demonstrating that they are subjected to hormonal control. Furthermore, experiments in isolated epithelial cells suggested that EZH2 is induced by progesterone in a paracrine fashion (Pal et al., 2013). Thus, changes in local tumor environment could alter methylation patterns and facilitate CSC formation in relatively few generations, as it does not require further mutations to occur.

## Factors influencing CSC formation

Selective pressure in a genetically unstable environment can drive selection for epigenetic or genetic changes that support survival. Factors that influence this tumor environment include infiltrating cells, hypoxia and chemotherapy, all of which have been linked to CSC development.

Co-culture of SUM159 cells with bone marrow-derived mesenchymal cells induced an expansion of the ALDH1-expressing SUM159 population (Liu et al., 2011). This expansion was due to a chemokine signaling loop between cancer-cell derived IL-6 and CXCL7 produced by ALDH^+^ mesenchymal cells. Moreover, co-injection of ALDH^+^ mesenchymal cells with SUM159 cells into NOD/SCID mice accelerated tumor growth and increased the capacity of the SUM159 cells to form secondary tumors following serial passage. Intratibial injection of mesechymal cells demonstrated that they could augment tumor growth and home to the site of breast tumor xenografts (Liu et al., 2011).

The immune response in FVB mice to cells derived from tumors in a Her2/neu transgenic strain caused the outgrowth of Her2-negative tumors. This antigen loss effect was dependent on CD8^+^ T cells. Her2-negative tumor cells had reduced CD24 levels compared with the parental Her2-positive cells and were more mesenchymal in appearance and expression patterns. Moreover, these CD24^−/low^ cells were much more tumorigenic than controls suggesting that the CD8^+^ T cell-dependent immune response was inducing EMT in the cancer cells to generate CSCs (Santisteban et al., 2009).

### Hypoxia

As tumors develop, the requirement for oxygen increases, leading to regions of hypoxia. Hypoxia causes activation of hypoxia-inducible factors, HIFs, which enable to cells to adapt to the low-oxygen environment. Hypoxic culture conditions (1% O_2_) induced an increase in the ALDH1^+^ proportion in breast cancer cell lines (Conley et al., 2012). Moreover, CSCs were enriched in hypoxic regions of tumor xenografts compared with normoxic regions (Conley et al., 2012). Using cycles of hypoxia and re-oxygenation to model the tumor microenvironment, Louie and colleagues enriched for populations of MDA-MB-231 and BCM2 cells that were significantly more tumorigenic than the parental lines (Louie et al., 2010). The hypoxia-selected populations also had a greater proportion of CD44^+^CD24^−/low^ cells. The low oxygen levels may influence the progenitor-like state of CSCs, as hypoxia blocked differentiation in MCF10A cells, possibly by maintaining greater levels of histone acetylation (Vaapil et al., 2012).

### Chemotherapy

In addition to CSCs forming as a part of tumor progression, therapeutic intervention may contribute to CSC genesis. Anti-angiogenic agents sunitinib and bevacizumab, which induce hypoxia in tumors, increased the number of CSCs in breast cancer xenografts (Conley et al., 2012). The release of factors by dying tumor cells may also act to augment the CSC pool. Interleukin-8 (IL-8) levels increased in SUM159 breast cancer cells following treatment with chemotherapeutic docetaxel (Ginestier et al., 2010). Interestingly, IL-8 signaling via its receptor CXCR1 on CSCs can expand CSC numbers in breast cancer cell lines (Charafe-Jauffret et al., 2009).

Further to the dying tumor cells releasing CSC-promoting factors, chemotherapy could alter the cells intrinsic mechanisms of preventing EMT. ER can directly suppress the EMT-driver SLUG; therefore anti-estrogen therapies may promote CSC formation by inducing EMT (Ye et al., 2008). Clearly the benefits of anti-estrogen therapies, such as tamoxifen, in prolonging patient survival are unarguable, but it is possible that under certain circumstances, initial anti-estrogen treatment may predispose the patient to recurrence of the disease.

## Pathological significance of breast cancer stem cells

### Tumor aggressiveness

Since the discovery of breast CSCs, they have been touted as critical targets for the design of future therapeutics. However, it is important to understand how CSCs influence the pathology of breast cancer so that treatments can be targeted appropriately.

Different subtypes of breast cancer are associated with different prognoses; luminal cancers offer the best chance of long-term survival and basal, claudin-low and Her2-positive cancers offer a much shorter life expectancy. Gene set enrichment analysis demonstrated similarity between the expression profile of stem cells and basal-breast cancers (Pece et al., 2010). The proportion of cells expressing stem-cell markers was approximately 3–4-fold higher in poorly differentiated compared with well-differentiated breast tumors. TAM-resistant ER-positive breast cancers are more basal-like, showing reduced E-Cadherin expression, increased CD44 and NF-κB expression along with increased motility (Hiscox et al., 2009).

A CSC gene signature from comparative analysis of CD44^+^CD24^−^ sorted tumor cells and cancer mammospheres showed that this signature was associated with claudin-low breast cancers, suggesting that claudin-low tumors are enriched for CSCs (Creighton et al., 2009). Moreover, the expression profile of the CSC-regulator, FOXC2 was enriched in claudin-low tumors and cell lines (Hollier et al., 2013). Her2 expression has been shown to correlate with ALDH1 expression in human breast cancer. ALDH1 levels also correlated with poor clinical outcome and proved to be an independent prognostic marker (Ginestier et al., 2007; Morimoto et al., 2009). Together, these studies suggest a link between CSCs and the aggressiveness of the disease.

In inflammatory breast cancer (IBC), ALDH1 expression correlated with histological grade but interestingly not with the CD44^high^CD24^−^ phenotype (Ginestier et al., 2007). This may be due to differences in analyzing CD44 and CD24 expression by immunohistochemistry rather than FACS or that CD44/CD24 may not be suitable markers of CSCs in IBC. A second study using IHC to assess prognostic significance of CD44 and CD24 expression in breast cancer also failed to find a correlation between the CD44^high^CD24^−^ phenotype and tumor progression, although there was suggestion of a correlation with bone metastasis (Abraham et al., 2005). These discrepancies between FACS and IHC studies could be due to the different techniques employed or other factors, such as the source of the tumor cells being analyzed.

There is accumulating evidence that CSC are involved in the metastatic progression of breast cancer. This is particularly significant given that the majority of cancer deaths are due to secondary lesions that have disseminated from the initial tumor. Immunohistochemistry of breast cancer cells isolated from bone marrow using the CD44^high^CD24^−/low^ phenotype suggests that there may be a much greater proportion of CSCs in metastatic tumors compared with the primary site (Balic et al., 2006). In IBC models, CSCs isolated by ALDH activity were shown to mediate metastasis in both *in vitro* and xenograft studies (Ginestier et al., 2007). Moreover, detection of ALDH^+^ cells in tumors from IBC patients correlated with both early onset of metastasis and overall decreased survival (Ginestier et al., 2007). CSCs have also been proposed to alter tissue architecture by driving epithelial remodeling. This disruption of normal tissue structure could be another method by which CSCs contribute to metastasis (Parashurama et al., 2012).

### Cancer recurrence following therapy

Resistance of CSCs to chemotherapy/radiotherapy is a possible mechanism to explain breast cancer recurrence. CSCs are enriched following neoadjuvant chemotherapy suggesting that CSCs are more resistant to therapy than the bulk of the tumor (Yu et al., 2007; Li et al., 2008). Treatment of both SUM159 and SUM149 cells with chemotherapeutics (paclitaxel or 5-fluorouracil) led to enrichment in the proportion of stem-like cells (Gupta et al., 2011). CSC-like MCF7 cells were resistant to several commonly used chemotherapeutics (Adriamycin, Etoposide, 5-Fluorouracil cis-Platinum, and Methotrexate), although they were more sensitive to Taxol (Creighton et al., 2009; Sajithlal et al., 2010).

The association between EMT and CSCs is also relevant to chemo-resistance, as cells undergoing EMT are more resistant to chemotherapeutics (Li et al., 2009). Cells isolated from Her2-antigen loss tumors that had undergone EMT had upregulated expression of protein pumps associated with drug resistance (BCRP and PGP). Accordingly, these cells were protected from chemotherapeutics mitoxantrone and etoposide. The mesenchymal tumor cells also had increased levels of DNA repair enzymes and were resistant to ionizing radiation (Santisteban et al., 2009).

### Tumor maintenance

CSCs are often referred to as being responsible for “maintaining” the tumor. In some respects, this maintenance role is an extrapolation of data showing that CSCs can recapitulate tumors of heterogeneous cell types over several passages in immune-compromised mice. Few studies have examined whether elimination of CSCs actually causes spontaneous-regression in the primary setting, which could be expected if the CSCs were maintaining the tumor. Part of the reason for this, is the lack of models in which to test the maintenance of tumors by CSCs.

Seminal lineage tracing experiments in both the skin and intestine demonstrated that during early transformation the tissues retain a cellular hierarchy akin to the normal tissue (Driessens et al., 2012; Schepers et al., 2012). Notably, in contrast to benign skin tumors, squamous cell carcinomas had an increased proportion of CSC, which had reduced propensity to differentiate. These studies demonstrate that CSCs exist early in the tumorigenesis process, but does still not delineate whether these early CSCs are maintaining the tumor. In a mouse model of glioblastoma, Chen and colleagues demonstrated the presence of quiescent CSCs that could expand and re-populate the tumor following chemotherapy with temozxolomide (TMZ). Eradication of these CSCs using a thymidine kinase transgene and ganciclovir (GCV) significantly improved survival. Moreover, the tumors in the GCV treated mice had reduced levels of proliferation and were less invasive suggesting that the CSCs were in indeed maintaining the tumor progression (Chen et al., 2012).

## Therapeutic targets in CSCs

The growing evidence that CSCs contribute to cancer progression and recurrence shows that developing anti-CSC therapies will likely improve chances of long-term survival of cancer patients. A proof of principle for targeting CSCs has been demonstrated in AML where the anti-leukemia drug TDZD-8 selectively killed leukemia stem cells while not affecting normal hematopoietic stem and progenitor cells (Guzman et al., 2007).

Many of the pathways currently under investigation as potential therapeutic targets in CSCs have been shown to regulate normal stem and progenitor cells, so finding methods to selectively target the pathways in cancer will be critical. Two developmental pathways that have received much recent attention as cell fate regulators in the breast are Notch and Wnt (Gu et al., 2013; Meier-Abt et al., 2013; Regan Joseph et al., 2013; Šale et al., 2013). It is therefore not surprising that they may be therapeutic targets in CSCs. In a model of Notch1-driven mammary tumorigenesis, inhibition of Notch signaling induced tumor regression and reduced tumorsphere formation *in vitro* (Simmons et al., 2012). Upregulation of the Notch ligand, Jagged2 in breast cancer cells and bone marrow derived cells in response to hypoxia led to an expansion of CSCs (Xing et al., 2011). Notch 4 activity is increased in breast CSCs and Notch and Wnt signaling were found to mediate radio-resistance in breast progenitor and CSCs (Phillips et al., 2006; Woodward et al., 2007; Harrison et al., 2010). The Wnt co-activator Pygo2 augmented mammosphere formation in MDA-MB-231 breast cancer cells (Chen et al., 2010). Conversely, deletion of pygo2 in MMTV-Wnt1 tumor cells reduced both mammosphere and tumor-forming capacity (Watanabe et al., 2013).

The potential therapeutic benefit of targeting Wnt-signaling was demonstrated by the identification of Salinomycin in a screen for CSC-inhibitors. Salinomycin preferentially eliminated CSCs by inhibiting Wnt signaling and inducing apoptosis Gupta et al., 2009; Fuchs et al., 2009; Lu et al., 2011; Tang et al., 2011. Salinomycin also killed iCSCL-10A cells that were resistant to Taxol and Actinomycin D (Nishi et al., 2013). Another drug that appears efficacious against CSCs is the anti-diabetic drug Metformin. Metformin targets CSC and can act synergistically with chemotherapy drugs to reduce CSC numbers and tumor growth (Hirsch et al., 2009; Vazquez-Martin et al., 2011). Subsequent work demonstrated that Metformin might act by inhibiting nuclear translocation of NF-κB and phosphorylation of STAT3 in CSCs compared with non-CSCs (Hirsch et al., 2013). Metformin may therefore be a candidate to treat TAM-resistant ER^+^ cancers that have been shown to upregulate NF-κB (Hiscox et al., 2009). Significantly, metformin treatment overcame Herceptin™ resistance in a Her2-positive xenograft model (Cufi et al., 2012).

Cell surface receptors make attractive targets for therapeutic design, as they are accessible to drugs. The growth factor receptor PDGFR-β was shown to lie downstream of FOXC2 in cells induced to undergo EMT and both proteins were expressed in CSC-enriched populations of SUM159 and HMLER cells (Hollier et al., 2013). The PDGFR-β inhibitor sunitinib reduced tumor growth and metastasis of FOXC2-expressing tumor cells (Hollier et al., 2013). Thus, sunitinib may be effective to combat CSC that arise as a result of EMT. FGF-receptor 2 (FGFR2) was enriched in CSC isolated from a MMTV-PyMT mouse breast cancer model (Kim et al., 2013). Moreover, FGFR2-expressing human tumor cells were more tumorigenic than FGFR2-negative cells in the xenograft experiments. Treatment with the FGFR inhibitor, TKI258, reduced the proportion of CSCs in MMTV-PyMT-driven tumors and delayed tumor growth (Kim et al., 2013).

The enrichment of CSCs that occurs under certain conditions, suggests that CSCs are capable of increasing their numbers by symmetric division. Blocking this mechanism of CSC expansion may slow tumor progression and allow more successful elimination of the CSC pool. By restoring p53 function in Her2 over-expressing cells, asymmetric cell division in the CSCs was restored leading to reduced tumor formation (Cicalese et al., 2009). Hedgehog (Hh) signaling via Bmi1 increased the frequency of mammosphere forming cells and this effect was reversed using the Hh inihibitor cyclopamine (Liu et al., 2006). Suppression of cFLIP eliminated CSCs in response to TRAIL, reducing formation of primary tumors in transplant models and almost completely preventing metastasis (Piggott et al., 2011). cFLIP suppression also reduced MFU-enrichment following passage of mammospheres, suggesting symmetric CSC division was compromised.

The plasticity of tumor cells is another hurdle that needs to overcome in order to prevent *de novo* CSC formation from non-CSCs. By blocking Activin/Nodal signaling, the ability of CD44^+^CD24^+^ (non-stem) cells to give rise to CD44^+^CD24^low^ (CSC) progeny was also blocked (Meyer et al., 2009).

Therapeutic ablation of specific cell populations is likely to only provide temporary relief from tumor progression. Moreover, as some therapies appear to support CSC production, it will be necessary to tackle cancer in a multi-pronged approach, targeting both CSC and non-CSCs. The CXCR1 inhibitor repertaxin killed bulk tumor cells by upregulating Fas expression and also prevented IL-8 signaling through CXCR1 to kill the CSCs (Ginestier et al., 2010). Combining GCV and TMZ to target both CSCs and non-CSCs significantly reduced the tumor burden compared with GCV treatment alone (Chen et al., 2012). Unfortunately, the outgrowth of cells that had suppressed the TK transgene precluded the authors from determining if there was a significant benefit to overall survival.

A problem with current cancer therapies is that they have been tested, selected and approved based on the ability to reduce tumor size without testing the effect on CSCs. Therefore, in addition to developing drugs that target CSCs it will be necessary to develop new assays focused on being able to detect changes in CSCs function that alone may not necessarily cause a reduction in tumor size. The efficacy of CSC-targeted therapeutics could also be determined by examining cancer recurrence in patients treated with combined drug regimes.

## Summary

There is now little doubt that cancer cells with the properties of stem cells exist within heterogeneous populations and that these CSCs have tumor-forming capacity. However, the role that these cells have in the formation and progression of the tumor in the primary setting is still unclear and will require suitable models to be developed for this to be delineated. The mechanisms of CSCs formation will require particular attention if they are to be successfully eliminated from patients. Finally, new assays that can detect the efficacy of targeting CSCs are essential if CSC-therapies are to make it to the clinic.

### Conflict of interest statement

The authors declare that the research was conducted in the absence of any commercial or financial relationships that could be construed as a potential conflict of interest.
